# Supplementary material for: Production and Assessment of Poly(Lactic Acid) Matrix Composites Reinforced with Regenerated Cellulose Fibres for Fused Deposition Modelling
Source: Polymers (Basel). 2022 Sep 23;14(19):3991. doi: 10.3390/polym14193991 (PMC9571083; doi:10.3390/polym14193991)
Supplement: Supplementary file 1 [file polymers-14-03991-s001.zip › polymers-1934301-supplementary.pdf]

Supplementary Information

Production and assessment of poly(lactic acid) matrix composites reinforced with regenerated cellulose fibres for fused deposition modelling

Christian Gauss [1,*], Kim L. Pickering [1], Joshua Tshuma[1] and John McDonald-Wharry[1]

School of Engineering, Division of Health, Engineering, Computing & Science, The University of Waikato, Private Bag 3105, Hamilton, New Zealand

* Correspondence: cgauss@waikato.ac.nz;

1. Supplementary information - Introduction

Table S1. Composition, production method and tensile properties of 3D printed PLA composites reinforced with cellulose-based fibres. Only the highest tensile properties given in the referenced publications are presented.

Filament Composition	Filament Production	Specimen type	UTS (MPa)	E (GPa)	ε_{break} (%)	Ref.
		**Nano cellulose**				
1 wt% Nano-fibrillated cellulose (NFC)	NFC dispersion in DMF is mixed with PLA dissolved in chloroform. Solvent was evaporated and the resulting powder was fed into a capillary rheometer at 180 °C to produce filaments	Printed (100% infill / ±45°)	41.2	3.37	2.1	[1]
1 wt% nano cellulose (NC)	NC dispersion in chloroform with dissolved polymer to obtain master batch of 10 wt. % NC. Mixed with neat PLA and extruded in a single-screw extruder at 100 - 190°C.	Filament	55.4*	3.91	4.5	[2]
		Printed (100% infill / ±45°)	28.4*	2.05	4.50	
2.5 wt% NFC	PEG60 mixed with PLA granules and NFC powder (1, 2.5 and 5 wt%) and extruded into filaments using a single-screw extruder at 170-175 °C.	Filament	58	-	4.50	[3]
30 wt% NFC	Dry NFC mixed with PLA dissolved in chloroform (10-40 wt%) and compounded in a Brabender melt compounder at 155 °C. Filaments produced through plunger-type batch extrusion.	Printed (100% infill / 0°)	~80	7.1	~1.5	[4]

Table S1. Continued.

Filament Composition	Filament Production	Specimen type	UTS (MPa)	E (GPa)	ε_{break} (%)	Ref.
		**Micro fibres**				
20 wt% thermomechanical pulp fibre (TMP)	Filaments produced by dual extrusion in a single-screw extruder using ground PLA and enzyme-treated TMP fibres (10-20%). Temperature profile of 175 – 165°C.	Filament	50.8	-	7.8	[5]
		Printed (100% infill / ±45º)	~22	-	-	
20 wt% Harakeke	10-30% of fibres compounded with PLA in a high shear mixer at 185 ºC and filaments produced in a twin-screw extruder at 150-185 ºC.	Printed (not specified)	~36	~4.2	-	[6]
10 wt% Hemp			~37	~3.5	-	
15 wt% Flax	PLA was compounded with two types of plasticizers sourced from Proviron Industries NV and 15 wt% of fibres in a twin-screw extruder at 165-200ºC.	Filament	~33	2.7	-	[7]
15 wt% bamboo fibre			~30	2.4		
20 wt% hemp fibre	Commercial PLA-Hemp filament – Hemprinted®	Not specified	33.6*	4.4	3.5	[8]

UTS – Tensile strength; E – Young's modulus; ε_{break} – Strain at break; DMF – Dimethylformamide; PEG – Polyethylene glycol. * Stress at break.

2. Representation of 3D printed samples

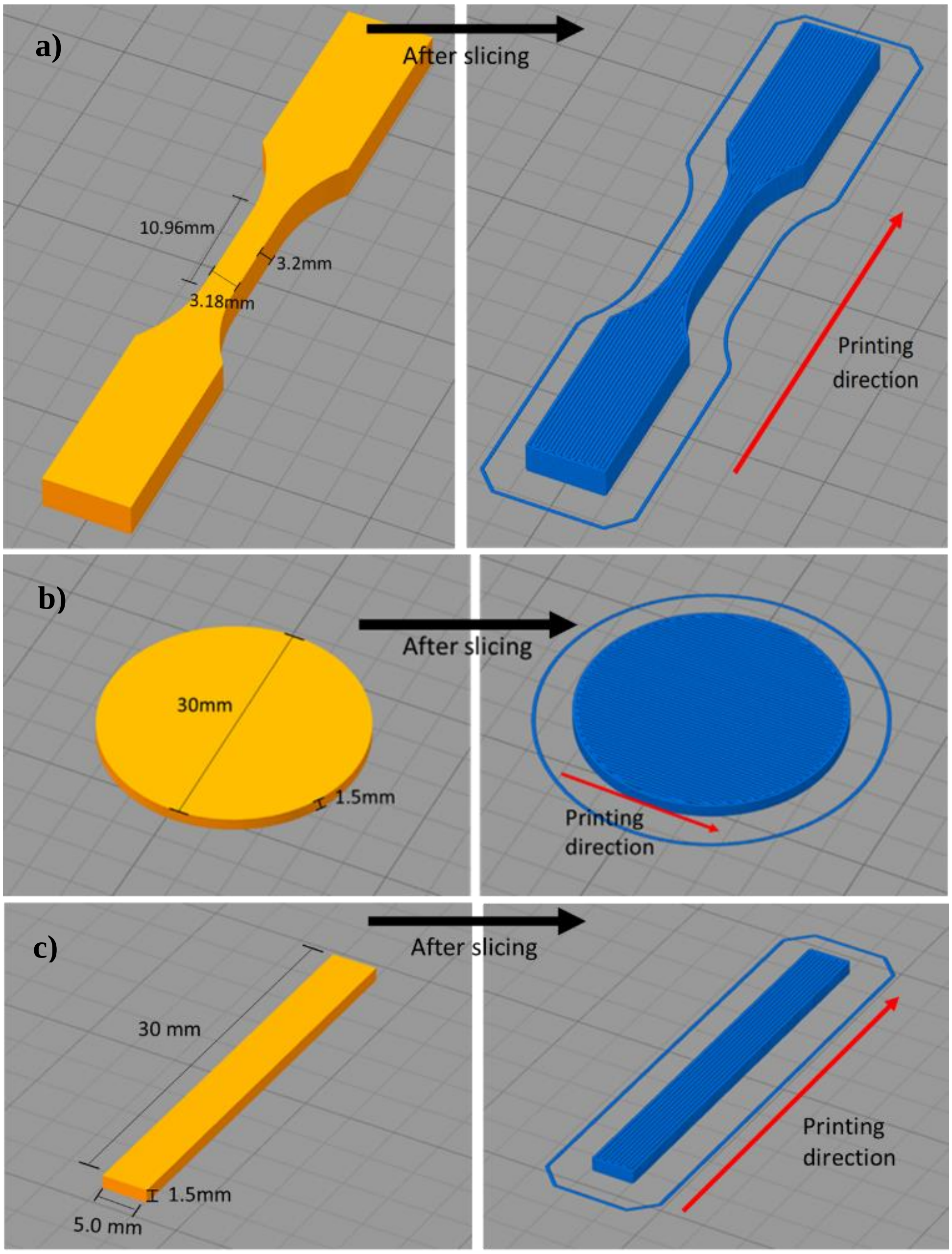

Figure S1. Dimensions and representation of printing path after slicing used to 3D print samples for tensile test (a), XRD (b), and DMA (c).

3. X-ray diffraction of lyocell fibres

The lyocell fibres were analysed in a Panalytical Empyrean XRD using CuKα radiation (40 kV; 40 mA) equipped with a PixCel linear detector. The fibres were scanned in a 2θ range of 5–45 ° using a scanning step of 0.01° and an equivalent exposure time of 40 s. The obtained X-ray diffraction pattern was analysed in the software HighScore® Plus (Panalytical) and submitted to a Rietveld refinement using the crystal structure of cellulose II [9]. The degree of crystallinity of cellulose was determined by including the amorphous phase during the refinement, as proposed by Nam et al. (2016) [10]. Crystallite size of planes ($10\bar{2}$) and (002) were calculated using the Scherrer equation [11].

Lyocell fibres have a cellulose II structure. This type of cellulose has a primitive monoclinic structure, with a crystal symmetry P 1 21 [12]. Figure S2 shows the experimental XRD pattern (in red) of the lyocell fibres used in this work and the calculated profile (as

the blue line) obtained by Rietveld refinement with the corresponding contributions of crystalline (green) and amorphous (purple) cellulose. The fibres presented a cellulose degree of crystallinity of 76.7 % and average crystallite size of 3.3 nm (calculated using the planes (110) and (020)). The degree of crystallinity of lyocell fibres is reported to be in the range of 64-80 %, which is higher than other forms of regenerated cellulose [13–15].

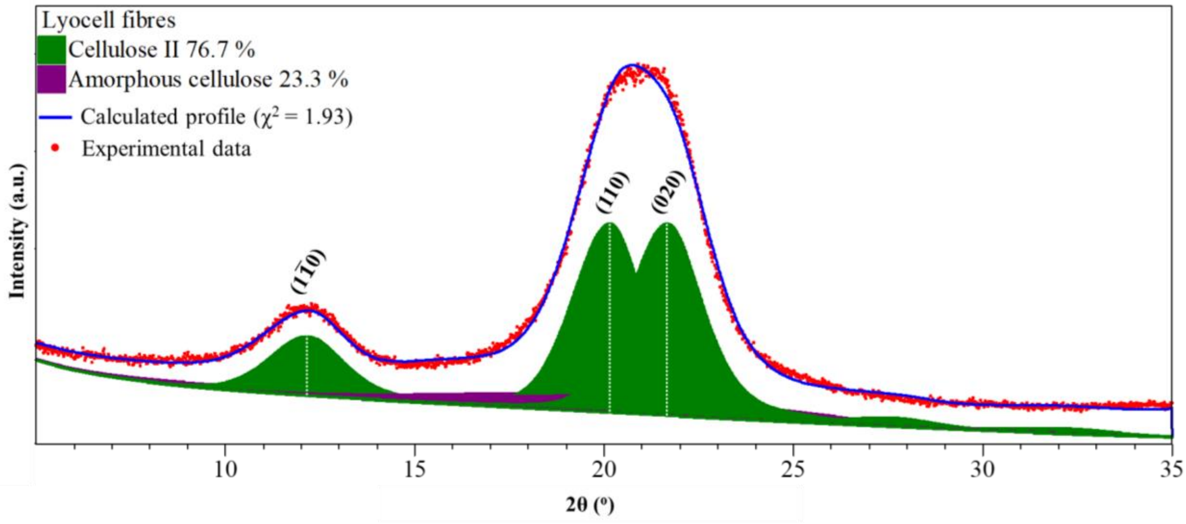

Figure S2. XRD diffraction pattern of lyocell fibres with corresponding calculated profile (in blue) using cellulose II structure.

4. Filaments characterisation

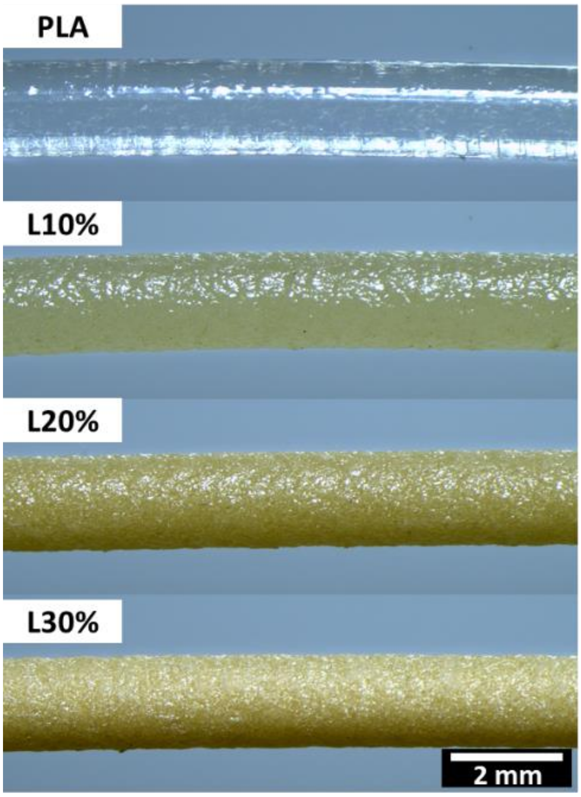

Figure S3. Stereo microscope images of the extruded filaments. From top to bottom: neat PLA, L10%, L20%, and L30%.

5. Characterisation of 3D printed samples

efore printing samples for tensile testing, DMA and XRD analyses, printability tests were conducted to adjust the printing parameters and obtain samples with satisfactory surface finish and dimensions. One of the parameters adjusted was the multiplier factor, which is a correction factor related to how much material is extruded during printing. Increasing the multiplier factor was found to decrease the dimensional accuracy of the samples and resulted in a higher deviation from the geometric specifications defined by the ASTM D638 (sample type V) standard. It also drastically reduced the surface quality of the specimen, leading to the requirement for sanding the faces of samples before testing. Low values of multiplier factor resulted in increased void content in samples which would affect the mechanical properties. An extrusion multiplier of 0.9 was selected as the most appropriate setting to use for 3D printing using the 3D printer used in this study. Figure S4 shows the resulting quality of a tensile test specimen printed with the L30% filament with an extrusion multiplier of 0.9. The surface quality of this specimen was also observed on all the other formulations.

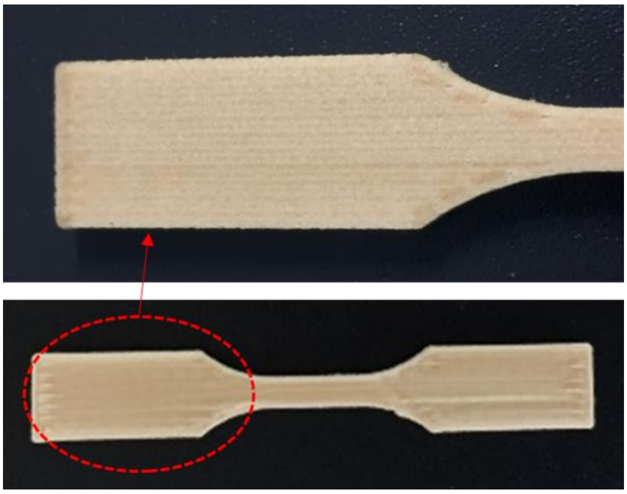

Figure S4. Close-up of the gripping area of 3D printed sample using adjusted multiplier factor.

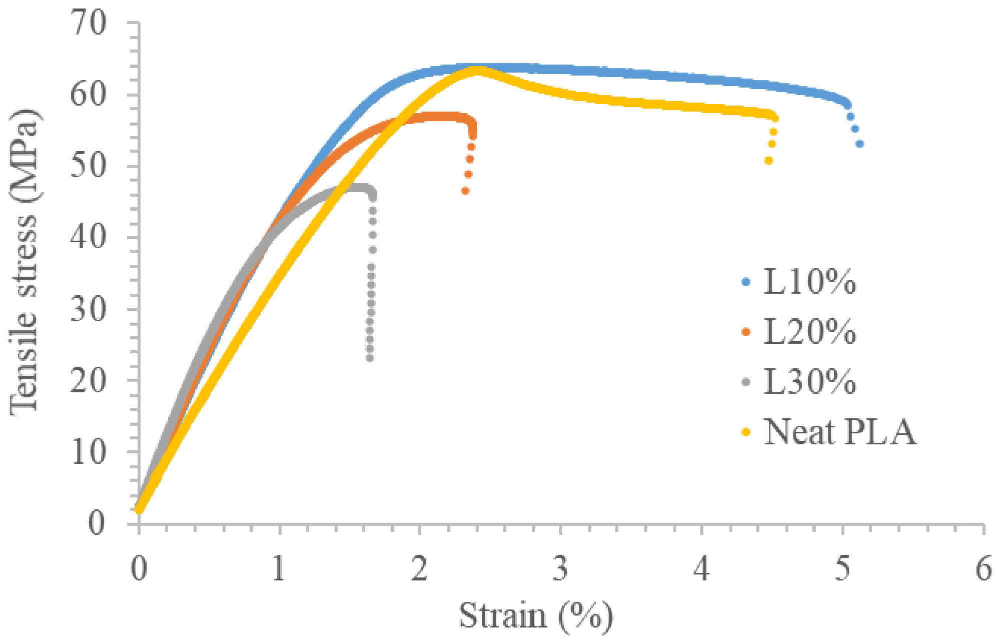

Figure S5. Tensile stress-strain curve of 3D printed samples of neat PLA and samples with 10-30% of lyocell fibres.

6. Fibre alignment

The shear rate experienced by the filament during extrusion can be defined according to Equation S1-S4 [16].

$$\dot{\gamma} = \frac{4Q}{\pi r^3} \quad \text{(S1)}$$

where Q is the flow rate and r is the radius of the nozzle. Considering that the flow rate is:

$$Q = \pi r^2 v \quad \text{(S2)}$$

where ◎ is the extrusion speed. Then:

$$\dot{\gamma} = \frac{4v}{r} \quad \text{(S3)}$$

In addition, the extrusion speed in the nozzle is proportional to the printing speed (speed of layer being deposited during printing) and corresponding nozzle size and layer dimensions (layer height and thickness). Therefore, it can be estimated as:

$$v = \frac{v_p(h_L w_L)}{\pi r^2} \quad \text{(S4)}$$

where v_P is the printing speed, and h_L and w_L are the layer height and width, respectively.

In XRD analysis, the orientation of cellulose fibres is observed in the change of diffracted intensity through the φ (azimuthal) axis. The degree of ordering (π) and Herman's order parameter (f) are used to determine the degree of cellulose alignment in each condition (determined using the reflection for cellulose II). The degree of ordering (π) and Herman's order parameter (f) can be calculated using Equations S5 and S6 [17]:

$$\pi = \frac{180 - FWHM}{180} \quad \text{(S5)}$$

where FWHM is the full-width half maximum of the analysed peak in an azimuthal scan.

$$f = \frac{3 < cos^2 l > -1}{2} \quad \text{(S6)}$$

where:

$$< cos^2 l >= 1 - 2 < cos^2\theta >$$

and

$$< cos^2\theta >= \frac{\int I(\varphi) cos^2\varphi sin\varphi d\varphi}{\int I(\varphi) sin\varphi d\varphi}$$

where θ and φ are the angles related to 2◎◎and◎azimuthal positions, respectively. A Herman's parameter of f = 1 corresponds to a maximum orientation, whereas f=0 indicates random orientation.

In Figure S6a, the obtained XRD patterns of 3D printed PLA samples with 20% of lyocell fibres at phi (ϕ)=0 º and 90 º in transmission mode are presented. The corresponding planes of cellulose II and the effect of fibre alignment can be observed. The difference in intensity in the peaks related to the (110)/(020) planes at different ϕ angles means that the lyocell fibres have a preferred orientation (caused by the printing process).

Figure S6b shows a phi scan of a sample with 20 % of fibres at 2θ = 21.4 º (plane (020) of cellulose II). The background has been subtracted for analysis purposes. The peaks centralised at ϕ = 90 and 270 º show that the (020) planes of the cellulose II crystallites are parallel to the printing direction.

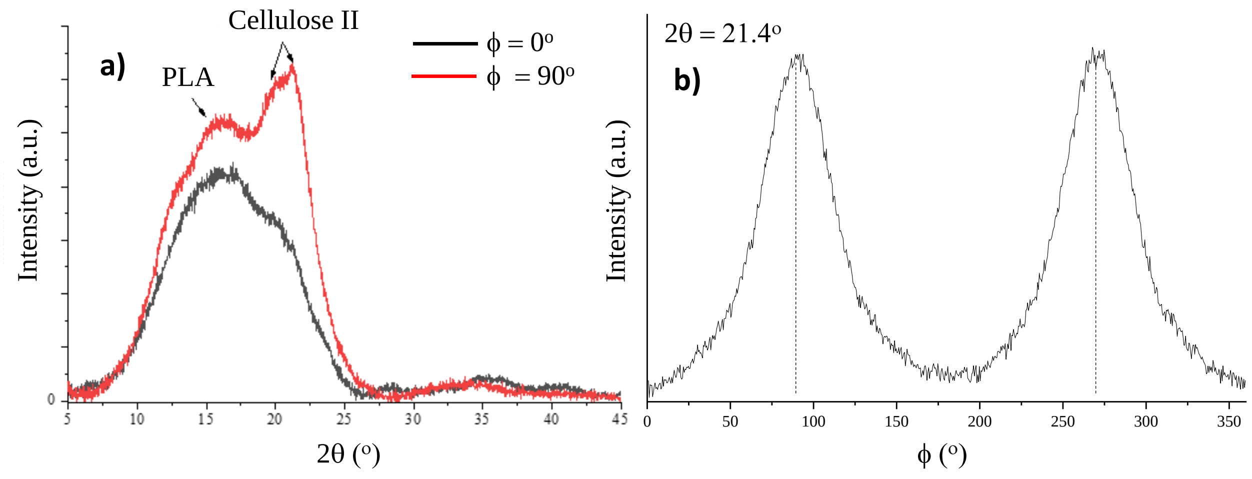

Figure S6. XRD patterns of 3D printed sample with 20% of fibres obtained by 2θ scan in transmission mode at φ angles of 0 ° and 90 ° (a) and azimuthal scan of the same sample showing the alignment of the fibres in the printing direction (b). The background was subtracted.

7. DMA samples

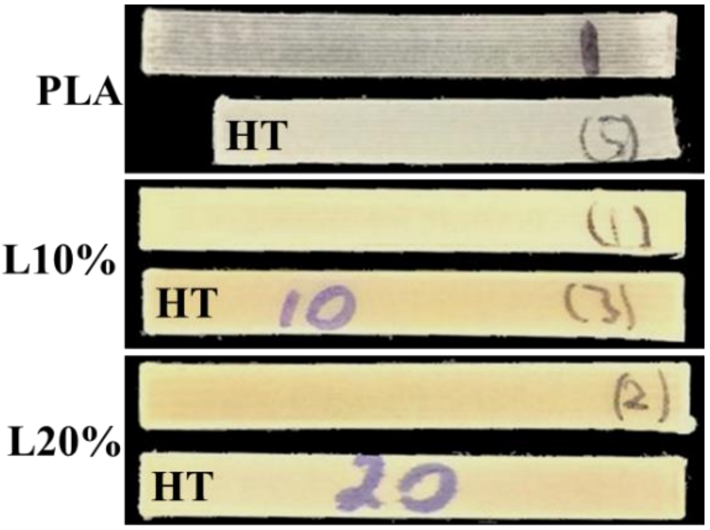

Figure S7. Effect of heat treatment on 3D printed samples of PLA, L10%, and L20% used for DMA analysis. HT = Heat treated at 105 °C for 2 h.

References

1. Ambone, T.; Torris, A.; Shanmuganathan, K. Enhancing the Mechanical Properties of 3D Printed Polylactic Acid Using Nanocellulose. *Polym. Eng. Sci.* **2020**, 1842–1855, doi:10.1002/pen.25421.
2. Rigotti, D.; Dorigato, A.; Cataldi, A.; Fambri, L.; Pegoretti, A. Nanocellulose as Reinforcing Agent for Biodegradable Polymers in 3D Printing Fused Deposition Modeling. *ECCM 2018 - 18th Eur. Conf. Compos. Mater.* **2020**, 24–28.
3. Wang, Q.; Ji, C.; Sun, L.; Sun, J.; Liu, J. Cellulose Nanofibrils Filled Poly(Lactic Acid) Biocomposite Filament for FDM 3D Printing. *Molecules* **2020**, *25*, 2319, doi:10.3390/molecules25102319.
4. Tekinalp, H.L.; Meng, X.; Lu, Y.; Kunc, V.; Love, L.J.; Peter, W.H.; Ozcan, S. High Modulus Biocomposites via Additive Manufacturing: Cellulose Nanofibril Networks as "Microsponges." *Compos. Part B Eng.* **2019**, *173*, 106817, doi:10.1016/j.compositesb.2019.05.028.
5. Filgueira, D.; Holmen, S.; Melbø, J.K.; Moldes, D.; Echtermeyer, A.T.; Chinga-Carrasco, G. Enzymatic-Assisted Modification of Thermomechanical Pulp Fibers to Improve the Interfacial Adhesion with Poly(Lactic Acid) for 3D Printing. *ACS*

Sustain. Chem. Eng. **2017**, *5*, 9338–9346, doi:10.1021/acssuschemeng.7b02351.

6. Stoof, D.; Pickering, K.; Zhang, Y. Fused Deposition Modelling of Natural Fibre/Polylactic Acid Composites. *J. Compos. Sci.* 2017, *1*.

7. Depuydt, D.; Balthazar, M.; Hendrickx, K.; Six, W.; Ferraris, E.; Desplentere, F.; Ivens, J.; Van Vuure, A.W. Production and Characterization of Bamboo and Flax Fiber Reinforced Polylactic Acid Filaments for Fused Deposition Modeling (FDM). *Polym. Compos.* **2019**, *40*, 1951–1963, doi:10.1002/pc.24971.

8. Kanesis Hemprinted Filament Technical Data Sheet - Natural Industrial Products Available online: https://www.hemprinted.com/wp-content/uploads/2021/05/TDS_HBP_SuperlabSRL_21112017.pdf.

9. French, A.D. Idealized Powder Diffraction Patterns for Cellulose Polymorphs. *Cellulose* **2014**, *21*, 885–896, doi:10.1007/s10570-013-0030-4.

10. Nam, S.; French, A.D.; Condon, B.D.; Concha, M. Segal Crystallinity Index Revisited by the Simulation of X-Ray Diffraction Patterns of Cotton Cellulose Iβ and Cellulose II. *Carbohydr. Polym.* **2016**, *135*, 1–9, doi:10.1016/j.carbpol.2015.08.035.

11. Scherrer, P. Bestimmung Der Inneren Struktur Und Der Größe von Kolloidteilchen Mittels Röntgenstrahlen. *Nachrichten von der Gesellschaft der Wissenschaften zu Göttingen* **1918**, *26*, 98–100.

12. Langan, P.; Nishiyama, Y.; Chanzy, H. X-Ray Structure of Mercerized Cellulose II at 1 Å Resolution. *Biomacromolecules* **2001**, *2*, 410–416, doi:10.1021/bm005612q.

13. Gindl-Altmutter, W.; Keckes, J.; Plackner, J.; Liebner, F.; Englund, K.; Laborie, M.P. All-Cellulose Composites Prepared from Flax and Lyocell Fibres Compared to Epoxy-Matrix Composites. *Compos. Sci. Technol.* **2012**, *72*, 1304–1309, doi:10.1016/j.compscitech.2012.05.011.

14. Colom, X.; Carrillo, F. Crystallinity Changes in Lyocell and Viscose-Type Fibres by Caustic Treatment. *Eur. Polym. J.* **2002**, *38*, 2225–2230, doi:10.1016/S0014-3057(02)00132-5.

15. Peng, S.; Shao, H.; Hu, X. Lyocell Fibers as the Precursor of Carbon Fibers. *J. Appl. Polym. Sci.* **2003**, *90*, 1941–1947, doi:10.1002/app.12879.

16. Giubilini, A.; Siqueira, G.; Clemens, F.J.; Sciancalepore, C.; Messori, M.; Nystrom, G.; Bondioli, F. 3D Printing Nanocellulose-Poly(3-Hydroxybutyrate-Co-3-Hydroxyhexanoate) Biodegradable Composites by Fused Deposition Modeling. *ACS Sustain. Chem. Eng.* **2020**, doi:10.1021/acssuschemeng.0c03385.

17. Yoshiharu, N.; Shigenori, K.; Masahisa, W.; Takeshi, O. Cellulose Microcrystal Film of High Uniaxial Orientation. *Macromolecules* **1997**, *30*, 6395–6397, doi:10.1021/ma970503y.
